# Supplementary material for: Determinants of long-term opioid use in hospitalized patients
Source: PLoS One. 2022 Dec 15;17(12):e0278992. doi: 10.1371/journal.pone.0278992 (PMC9754198; doi:10.1371/journal.pone.0278992)
Supplement: S1 File — (DOCX) [file pone.0278992.s001.docx]

Appendix A. Codes Used for Drug Classification

ATC codes used to identify opioids: N02A (opioids), R05DA (opium alkaloids and derivatives)

Exclusions: Not all drug forms were included in the analyses. Only patches and tablets of these medications were kept. Injectable, liquid and rectal forms were excluded. Methadone and buprenorphine/naloxone combinations were kept to define subclinical patient populations but were excluded from all dosing/duration calculations as these medications are used to treat addiction and we want to focus on the association of duration/dose of opioids used for pain relief.

Appendix A.1. Opioid Morphine Equivalent Conversion Factor **^1^**

**Drug Name Conversion Factor**

| Buprenorphine patch^2^ | 12.6 |
| --- | --- |
| Buprenorphine tab or film | 10 |
| Butorphanol | 7 |
| Codeine | 0.15 |
| Dihydrocodeine | 0.25 |
| Fentanyl buccal or SL tablets, or lozenge/troche^3^ | 0.13 |
| Fentanyl film or oral spray^4^ | 0.18 |
| Fentanyl nasal spray^5^ | 0.16 |
| Fentanyl patch^6^ | 7.2 |
| Hydrocodone | 1 |
| Hydromorphone | 4 |
| Levorphanol tartrate | 11 |
| Meperidine hydrochloride | 0.1 |
| Methadone | 3 |
| Morphine | 1 |
| Nalbuphine | 1 |
| Opium | 1 |
| Oxycodone | 1.5 |
| Oxymorphone | 3 |
| Pentazocine | 0.37 |
| Tapentadol | 0.4 |
| Tramadol | 0.1 |

^1^ Centers for Disease Control and Prevention, Atlanta, GA, May 2014.

^2^ The MME conversion factor for buprenorphine patches is based on the assumption that one milligram of parenteral buprenorphine is equivalent to 75 milligrams of oral morphine and that one patch delivers the dispensed micrograms per hour over a 24-hour day. Example: 5 ug/hr buprenorphine patch * 24 hrs = 120 ug/day buprenorphine = 0.12 mg/day buprenorphine = 9 mg/day oral morphine milligram equivalent. In other words, the conversion factor not accounting for days of use would be 9/5 or 1.8. However, since the buprenorphine patch remains in place for 7 days, we have multiplied the conversion factor by 7 (1.8 X 7 = 12.6). In this example, MME/day for four 5 μg/hr buprenorphine patches dispensed for use over 28 days would work out as follows: Example: 5 ug/hr buprenorphine patch * (4 patches/28 days) * 12.6 = 9 MME/day.

^3^ The MME conversion factor for fentanyl buccal tablets, sublingual tablets, and lozenges/troche is 0.13. This conversion factor should be multiplied by the number of micrograms in a given lozenge/troche.

^4^ The MME conversion factor for fentanyl film and oral spray is 0.18. This reflects a 40% greater bioavailability for films compared to lozenges/tablets and 38% greater bioavailability for oral sprays compared to lozenges/tablets.

^5^ The MME conversion factor for fentanyl nasal spray is 0.16, which reflects a 20% greater bioavailability for sprays compared to lozenges/tablets.

| ^6^ The MME conversion factor for fentanyl patches is based on the assumption that one milligram of parenteral fentanyl  is equivalent to 100 milligrams of oral morphine and that one patch delivers the dispensed micrograms per hour over a  24 hour day. Example: 25 ug/hr fentanyl patch * 24 hrs = 600 ug/day fentanyl = 60 mg/day oral morphine milligram  equivalent. In other words, the conversion factor not accounting for days of use would be 60/25 or 2.4. However, since  the fentanyl patch remains in place for 3 days, we have multiplied the conversion factor by 3 (2.4 X 3 = 7.2).  In this example, MME/day for ten 25 μg/hr fentanyl patches dispensed for use over 30 days would work out as  follows: Example: 25 ug/hr fentanyl patch * (10 patches/30 days)* 7.2 = 60 MME/day. |
| --- |

Sources:

1. Centers for Medicare & Medicaid Services. Opioid Oral Morphine Milligram Equivalent (MME) Conversion Factors. https:\\www.cms.govMedicarePrescription-Drug-CoveragePrescriptionDrugCovContraDownloadsOpioid-Morphine-EQConversion-Factors-vFeb-.pdf. Accessed: September 5, 2019
2. Svendsen, K., Borchgrevink, P., Fredheim, O., Hamunen, K., Mellbye, A., & Dale, O. (2011). Choosing the unit of measurement counts: the use of oral morphine equivalents in studies of opioid consumption is a useful addition to defined daily doses. Palliative Medicine, 25(7), 725–732. <http://doi.org/10.1177/0269216311398300>

Appendix A.2. Long-term Opioid Use Construction

Opioid use in the one-year post-discharge period was ascertained using RAMQ pharmacy administrative claims. For each prescription filled, these claims document the specific medication using the drug identification number (DIN), strength, dispensing date and quantity, duration of the prescription, and prescribing physician. DINs that mapped to Anatomical Therapeutic Chemical Classification System (ATC) codes N02A, R05DA were used to identify opioids (see Appendix 5-A for opioid inclusion criteria and dose calculation). Duration of opioid use was based on the number of days of medication supplied in each dispensed prescription. A drug-by-day matrix was created for each patient, for the 12 months following discharge, using the date and duration of each opioid prescription. On each day, an individual was classified as having a dispensed supply of an opioid available or not.

Appendix B. Inclusion of Covariates and Their Assessment in the Cox Proportional Hazards Models

­­Appendix B.1. Description of available data on drug, patient, provider and system level characteristics

|  | **Description** | **Measurement** | **Timing of Measurement** | **Functional Form** |
| --- | --- | --- | --- | --- |
| **Opioid-related Characteristics** | | | | |
| ***Opioid Dispensations*** | | | | |
| ATC code | Anatomical Therapeutic Chemical Classification System code used to identify opioids and other concurrent medications that the patient is taking  **Opioids ATC Included: N02A, R05DA** | RAMQ prescription claims. | In the community one year prior to admission and one year post-discharge | N/A |
| Dose | The daily amount of drug taken by patient will be calculated based on information about the number of tablets prescribed, strength and number of days’ supply; daily dose will be converted to milligram morphine equivalents to facilitate comparisons across opioids. | From RAMQ prescription claims | In the community one year prior to admission and one year post-discharge | Continuous, categorical, time-varying |
| Duration | The days’ supply on the drug claim as entered by the pharmacist | From RAMQ prescription claims | In the community one year prior to admission and one year post-discharge | Continuous, categorical, time-varying |
| Type of opioid | Type of opioid ingredient. E.g; Hydromorphone, oxycodone, morphine, fentanyl, etc. | From RAMQ prescription claims | One year post-discharge | Categorical, time-varying |
| ***Opioid Administration in Hospital*** | | | | |
| ATC code | Anatomical Therapeutic Chemical Classification System code used to identify administered opioids | Hospital pharmacy | In hospital | Categorical |
| ***Opioid Prescription at Hospital Discharge*** | | | | |
| Status of opioid medication | Continued or stopped from community, newly prescribed at discharge | From patient chart | At hospital discharge | Categorical, time-fixed |
| Reason for opioid prescribing | Pain-related including having had surgery as well as other diagnoses such as having a cancer or a pain syndrome diagnose | From patient chart | In-hospital | Categorical,  time-fixed |
| Presence of a multi-modal pain management regimen | The opioid prescription at hospital discharge was part of multi-modal pain treatment regimen | From patient chart | At hospital discharge | Categorical,  time-fixed |
| **Patient-level Characteristics** | | | | |
| ***Demographics*** |  |  |  |  |
| Age |  | From patient chart | Admission to hospital | Continuous, time-varying |
| Sex | Male, Female | From patient chart | Admission to hospital | Binary, time-fixed |
| Drug insurance status | E.g.; Full copay, partial copay, no copay Serves as proxy for socio-economic status. | From RAMQ drug programs | Admission to hospital | Categorical, time-fixed |
| ***Co-Existing Illnesses*** | | | | |
| History of mental health conditions | E.g.; Anxiety, depression, psychiatric diagnosis, mood disorder, and post-traumatic stress disorder | ICD-9 from RAMQ medical services and ICD-10 codes from hospitalization data | In community one year prior to admission, in hospital, post-discharge | Binary per condition, time-varying |
| Pain syndromes | E.g.; Chronic back pain, back and neck pain, back disorder, arthritis, migraine, headache, fibromyalgia, fracture | ICD-9 from RAMQ medical services and ICD-10 codes from hospitalization data | In community one year prior to admission, in hospital, post-discharge | Binary per condition, time-varying |
| Health conditions Associated with abuse | E.g.; Alcohol abuse, drug abuse | From patient chart. Also from RAMQ medical series and prescription claims | In community one year prior to admission, in hospital, post-discharge | Binary per condition, time-varying |
| Cancer diagnosis | E.g.; Metastatic, Non-metastatic, Lymphoma | ICD-9 from RAMQ medical services and ICD-10 codes from hospitalization data | In community one year prior to admission, in hospital, post-discharge | Binary per condition, time-varying |
| Other comorbidities | E.g.; Acute MI, cerebrovascular diseases, chronic kidney, COPD, diabetes, heart failure, hypertension, ischemic heart disease, liver, obesity | ICD-9 from RAMQ medical services and ICD-10 codes from hospitalization data | In community one year prior to admission, in hospital, post-discharge | Binary per condition, time-varying |
| ***Drug and Healthcare Utilization*** | | | | |
| Use of potential interacting drugs increasing the risk of opioid misuse | E.g.; Selective serotonin reuptake inhibitors, other antidepressants, benzodiazepines, other antipsychotic drugs, central nervous system depressants, psychotropic medication | ATC codes, DIN, Generic Drug name used to extract information from RAMQ prescription claims, hospital data, patient chart. | In community one year prior to admission, in-hospital, post-discharge | Binary per drug, time-varying |
| Use of non-opioid pain medications | E.g.; NSAIDS, COX-2, Acetaminophen, Gabapentin, anti-migraine medications, muscle-relaxants, other anti-inflammatories and anti-rheumatoid medications | ATC codes, DIN, Generic Drug name used to extract information from RAMQ prescription claims, hospital data, patient chart. | In community one year prior to admission, in-hospital, post-discharge | Binary per drug, time-varying |
| Number of ED visits and hospitalizations | Total number of ED visits and hospitalizations | From RAMQ prescription claims and hospital data | One year prior to hospital admission & one year post-discharge | Categorical, continuous, time-varying |
| Number of physicians | Number of unique prescribing physicians | From RAMQ medical services | One year prior to hospital admission & one year post-discharge | Categorical, continuous, cumulative time-varying |
| ***Other Patient Drug Behavior Characteristics*** | | | | |
| Time since hospital discharge | The time elapsed between patent’s hospital discharge and their first opioid dispensation | From RAMQ prescription claims and hospital data | One year post-discharge | Continuous, time-fixed |
| Add-on opioid | Recent add-on of another opioid type in the past 2 weeks | From RAMQ medical services | One year post-discharge | Categorical, time-varying |
| **In-hospital Characteristics** | | | | |
| Hospital patient is admitted to | Montreal General or Royal Victoria hospital | From hospital chart | Upon admission to the hospital | Binary, time-fixed |
| Hospital unit the patient is admitted to | Medical or surgical unit | From hospital chart | Upon admission to the hospital | Binary, time-fixed |
| Discharge destination | Home community, long term care | From hospital chart | Upon discharge | Binary, time-fixed |
| **Attending Physician Characteristics** | | | | |
| Years of practice | Number of years practiced since graduation from medical school | From hospital chart, Collège des médecines du Quebec | Upon discharge | Categorical, time-fixed |
| Sex | Male, Female | From hospital chart | Upon discharge | Binary, time-fixed |
| Language | Home community, long term care | From hospital chart | Upon discharge | Binary, time-fixed |
| Discharge prescription signed by | Attending physician vs resident | From hospital chart | Upon discharge | Binary, time-fixed |

Appendix C. Sensitivity analyses

Appendix C. 1. Sensitivity analyses including selected time-varying characteristics in the model.

|  |  | **HR** | **95% CI** |
| --- | --- | --- | --- |
| Non-opioid pain medications use |  |  |  |
| No |  | Reference | Reference |
| Yes |  | 1.39 | 0.97 – 1.82 |
| Benzodiazepine use |  |  |  |
| No |  | Reference | Reference |
| Yes |  | 0.81 | 0.44 – 1.48 |
| Antidepressant use |  |  |  |
| No |  | Reference | Reference |
| Yes |  | 1.67 | 1.16 – 2.41 |
| Mental illness/substance & alcohol abuse |  |  |  |
| No |  | Reference | Reference |
| Yes |  | 0.87 | 0.66 – 1.15 |
| Pain syndromes |  |  |  |
| No |  | Reference | Reference |
| Yes |  | 1.21 | 0.95 – 1.53 |
| Cancer |  |  |  |
| No |  | Reference | Reference |
| Yes |  | 1.39 | 1.07 – 1.81 |

Appendix C. 2. Sensitivity analyses including time-varying count of distinct prescribing physicians and an indicator for using ≥2 opioid products in the model.

|  |  | **HR** | **95% CI** |
| --- | --- | --- | --- |
| MME Dose |  |  |  |
| ≤20 |  | Reference | Reference |
| 20-50 |  | 1.10 | 0.82 – 1.49 |
| 50-90 |  | 0.97 | 0.64 – 1.48 |
| >90 |  | 2.91 | 1.62 – 5.23 |
| Initial days’ supply |  |  |  |
| ≤7 |  | Reference | Reference |
| >7 |  | 1.34 | 1.05 – 1.72 |
| Prescribing physicians |  |  |  |
| 0-1 |  | Reference | Reference |
| 2-3 |  | 2.43 | 1.85 – 3.19 |
| ≥4 |  | 5.95 | 4.33 – 8.18 |
|  |  |  |  |

Appendix C. 3. Sensitivity analyses including selected time-varying characteristics, distinct prescribing physicians and an indicator for using ≥2 opioid products in the model.

|  |  | **HR** | **95% CI** |
| --- | --- | --- | --- |
| Non-opioid pain medications use |  |  |  |
| No |  | Reference | Reference |
| Yes |  | 1.38 | 1.01 – 1.87 |
| Benzodiazepine use |  |  |  |
| No |  | Reference | Reference |
| Yes |  | 0.81 | 0.44 – 1.48 |
| Antidepressant use |  |  |  |
| No |  | Reference | Reference |
| Yes |  | 1.67 | 1.15 – 2.39 |
| Mental illness/substance & alcohol abuse |  |  |  |
| No |  | Reference | Reference |
| Yes |  | 0.89 | 0.68 – 1.18 |
| Pain syndromes |  |  |  |
| No |  | Reference | Reference |
| Yes |  | 1.07 | 0.84 – 1.36 |
| Cancer |  |  |  |
| No |  | Reference | Reference |
| Yes |  | 1.06 | 0.81 – 1.39 |
| Prescribing physicians |  |  |  |
| 0-1 |  | Reference | Reference |
| 2-3 |  | 2.41 | 1.83 – 3.17 |
| ≥4 |  | 5.99 | 4.34 – 8.26 |

Appendix C.4. The association between patient, medication and system-level characteristics and time to long term use within the one-year post-discharge among opioid-naïve users (n=1050)

|  |  | **Hazard**  **Ratio** | **95% CI** |  |
| --- | --- | --- | --- | --- |
| **Drug copay status** |  |  |  |  |
| Full |  | Reference | Reference |  |
| Partial |  | 1.27 | 0.80 – 2.01 |  |
| None |  | 2.25 | 1.46 – 3.47 |  |
| **Benzodiazepine use** |  |  |  |  |
| No use |  | Reference | Reference |  |
| Use |  | 1.70 | 1.15 – 2.51 |  |
| **Cancer diagnoses** |  |  |  |  |
| No use |  | Reference | Reference |  |
| Use |  | 1.42 | 0.96 – 2.10 |  |
| **Pain syndromes** |  |  |  |  |
| No use |  | Reference | Reference |  |
| Use |  | 1.54 | 1.01 – 2.23 |  |
| **Pain Regimen** |  |  |  |  |
| *Opioid prescription coming from the*  *in-hospital prescriber* | |  |  |  |
| No |  | Reference | Reference |  |
| Yes |  | 0.59 | 0.39 – 0.91 |  |
| **MME Dose** |  |  |  |  |
| ≤20 |  | Reference | Reference |  |
| 20-50 |  | 0.90 | 0.75 – 1.40 |  |
| 50-90 |  | 1.06 | 0.56 – 2.01 |  |
| >90 |  | 6.99 | 2.05 – 23.8 |  |

**Note:** All results obtained using a Cox Proportional Hazards Model. All variables were included in the model.

Appendix C.5. The association between patient, medication and system-level characteristics and time to long term use within the one-year post-discharge among previous opioid users (n=192)

|  |  | **Hazard**  **Ratio** | **95% CI** |  |
| --- | --- | --- | --- | --- |
| **Drug copay status** |  |  |  |  |
| Full |  | Reference | Reference |  |
| Partial |  | 2.03 | 1.13 – 3.64 |  |
| None |  | 2.05 | 1.14 – 3.70 |  |
| **Benzodiazepine use** |  |  |  |  |
| No use |  | Reference | Reference |  |
| Use |  | 1.96 | 1.19 – 3.23 |  |
| **Non-opioid medications use** |  |  |  |  |
| No use |  | Reference | Reference |  |
| Use |  | 1.76 | 1.08 – 2.88 |  |
| **Surgery during the index admission** |  |  |  |  |
| No |  | Reference | Reference |  |
| Yes |  | 0.35 | 0.21 – 0.61 |  |
| **Pain Regimen** |  |  |  |  |
| *Opioid prescription coming from the*  *in-hospital prescriber* | |  |  |  |
| No |  | Reference | Reference |  |
| Yes |  | 0.61 | 0.36 – 1.01 |  |
| **MME Dose** |  |  |  |  |
| ≤20 |  | Reference | Reference |  |
| 20-50 |  | 1.14 | 0.66 – 1.97 |  |
| >50 |  | 1.89 | 0.87 – 4.13 |  |
| **Days’ Supply** |  |  |  |  |
| ≤7 |  | Reference | Reference |  |
| >7 |  | 2.62 | 1.52– 4.53 |  |

**Note:** All results obtained using a Cox Proportional Hazards Model. All variables were includein the model.

Appendix D. Empirically-defined threshold for long-term opioid therapy.

Appendix D.1. Comparison of goodness of fit of flexible marginal structural models, with alternative time-varying opioid exposure metrics**.**

| **Opioid Exposure Metric** | **Statistical Model** | | **AIC** |
| --- | --- | --- | --- |
| ***Cumulative Use*** |  | |  |
|  | Conventional Cox MSM | | 2606.3 |
|  | Flexible non-linear (NL) MSM | | 2584.0 |
| ***Continuous Use*** |  | |  |
|  | Conventional Cox MSM | | 2611.4 |
|  | | Flexible non-linear (NL) MSM | 2596.0 |
|  | |  |  |

Appendix D.1a Non-linear effect of ***duration of*** ***cumulative opioid use*** truncated at 180 days and the risk of opioid-related emergency department visits, re-admissions or deaths.

Appendix D.1b. Operational definition of long-term opioid use therapy in the presence/absence of post-baseline hospitalizations.


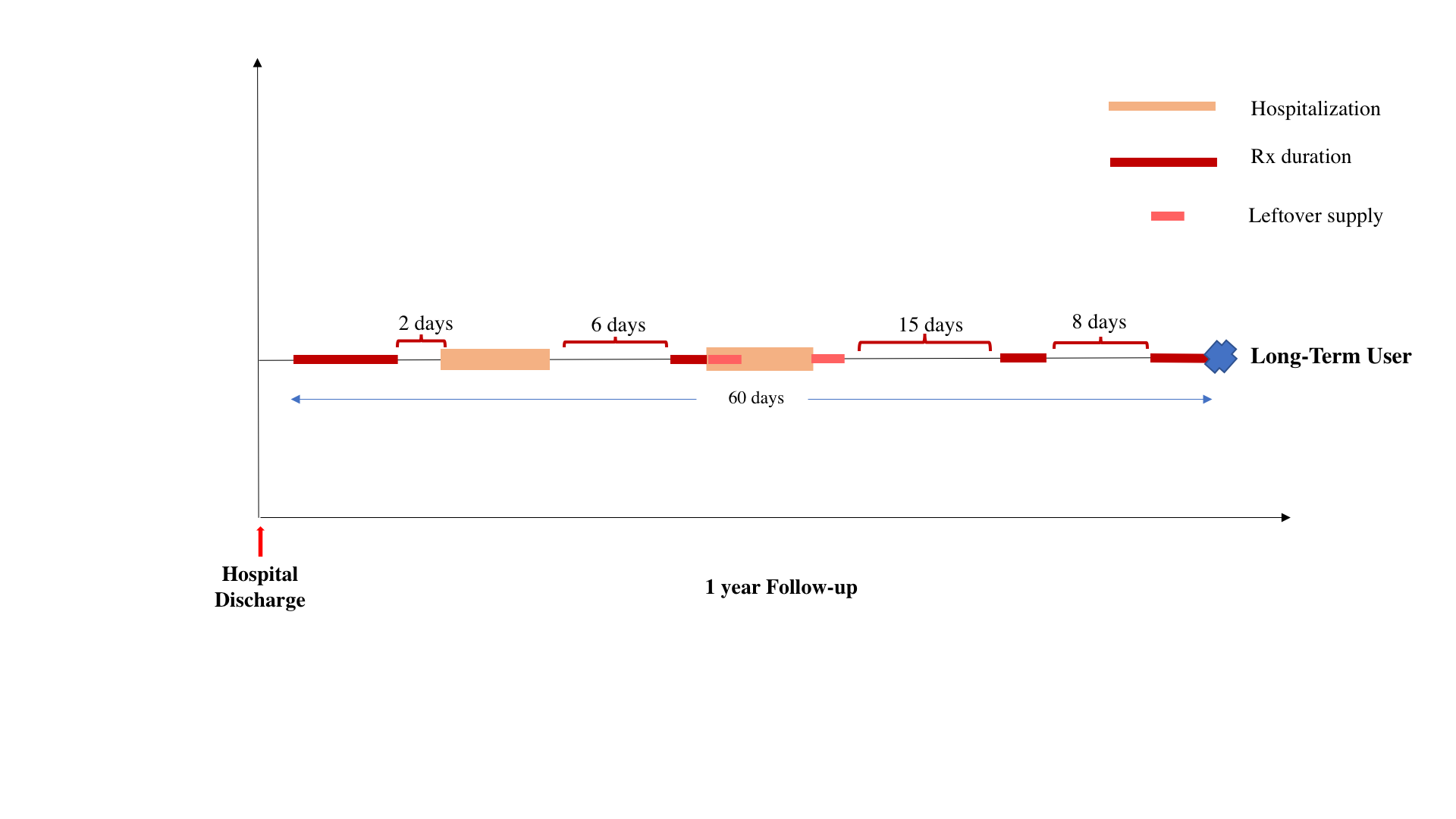


Appendix E. Results from assessing the proportional hazards (PH) assumption in the main Cox PH model.

| Covariate | p-value |
| --- | --- |
| Age (>64) | 0.94 |
| Female | 0.96 |
| Drug copay status “partial” | 0.62 |
| Drug copay status “full” | 0.034 |
| Emergency department visits/hospitalizations ≥1 | 0.62 |
| Episodic opioid use 1-60 days | 0.96 |
| Long-term opioid use ≥ 60 days | 0.16 |
| Benzodiazepine use | 0.58 |
| Antidepressant use | 0.85 |
| Non-opioid medication use | 0.07 |
| Mental illness/substance and alcohol use | 0.88 |
| Charlson Comorbidity Index 1-2 | 0.81 |
| Charlson Comorbidity Index ≥3 | 0.23 |
| In-hospital opioid use | 0.94 |
| In-hospital non-opioid use | 0.46 |
| Cardiothoracic surgery | 0.28 |
| Gastrointestinal surgery | 0.21 |
| Thoracic surgery | 0.51 |
| Unrelated | 0.40 |
| Opioid prescription at discharge | 0.83 |
| Cancer treatment indication | 0.049 |
| Pain syndromes treatment indication | 0.80 |
| Attending physician years of practice 20-40 | 0.055 |
| Attending physician years of practice >40 | 0.71 |
| Attending physician female | 0.19 |
| Attending physician language French | 0.097 |
| Discharge prescription signed by resident | 0.13 |
| Hospital discharge destination long-term care facility | 0.062 |
| Opioid dispensed codeine | 0.83 |
| Opioid dispensed hydromorphone | 0.18 |
| Opioid dispensed morphine | 0.62 |
| Opioid dispensed oxycodone | 0.62 |
| Opioid dispensed fentanyl | 0.016 |
| Dose dispensed 20-50 MME | 0.43 |
| Dose dispensed 50-90 MME | 0.54 |
| Dose dispensed >90 MME | 0.010 |
| Days’ supply dispensed >7 | 0.89 |

Appendix E.1. Smooth residual plot for the time-dependent effect of the initial opioid dose dispensation being greater than 90MME.


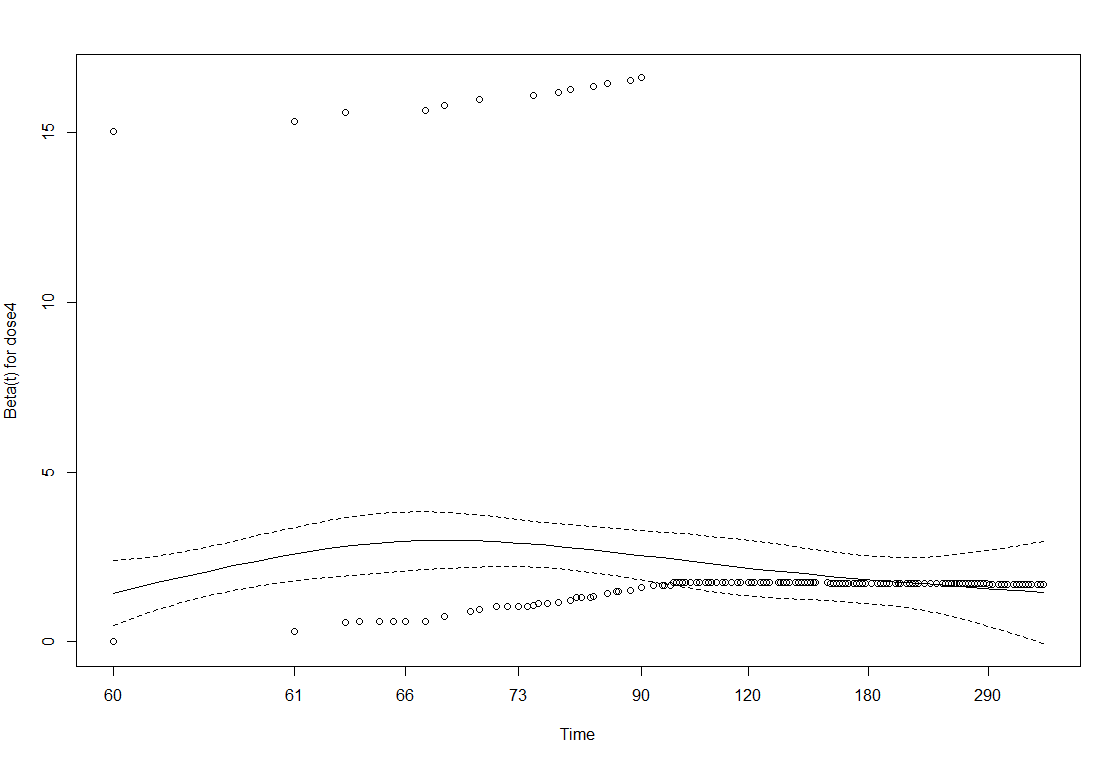


Supplemental Material E.1.

From the graph of the smooth residual plot, we see that initial dose dispensed of >90MME is associated with an increase in risk over the entire follow-up period. This consistent increase in risk is observed under constant proportional hazards effect with a p-value of 0.01 (Appendix E) and a HR >1 (HR=2.08). This increase in risk of the outcome associated with an initial dispensed dose of >90MME were even higher during the first three to four months of follow-up.

Appendix E.2. Smooth residual plot for the time-dependent effect of having fentanyl as the initial opioid dispensation.


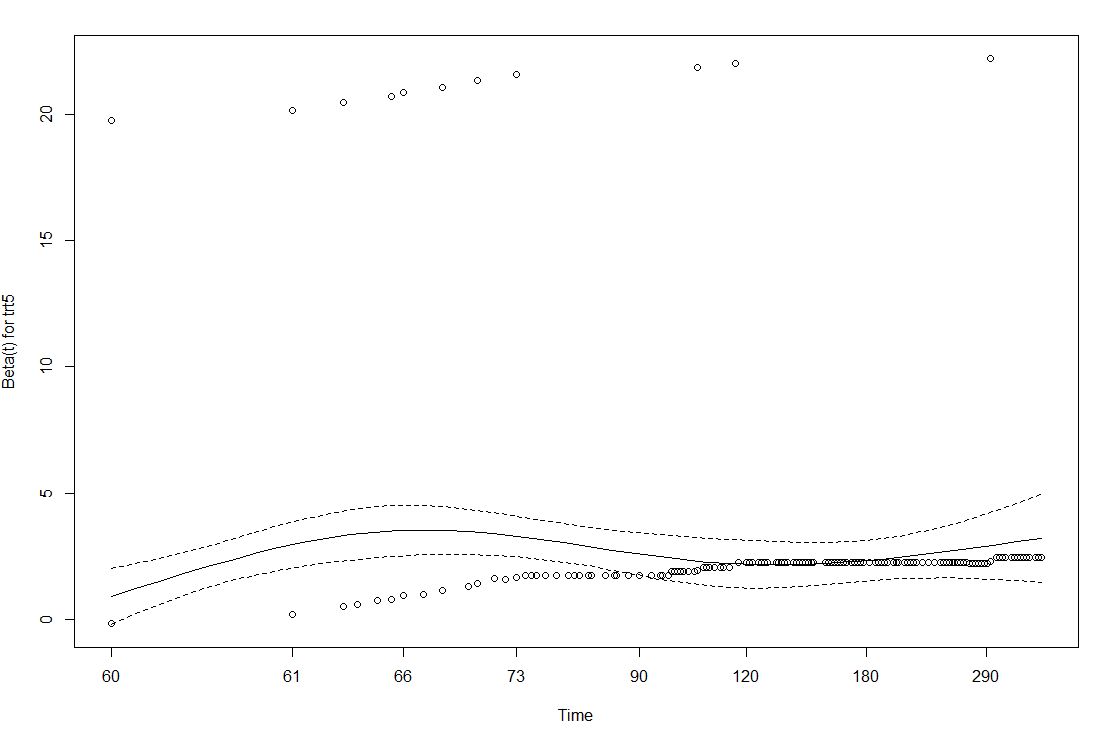


Supplemental Material E.2.

From the graph of the smooth residual plot, we see that initial dispensed of fentanyl is associated with an increase in risk over the entire follow-up period. This consistent increase in risk is observed under constant proportional hazards effect with a p-value of 0.016 (Appendix E).

Appendix E.3. Smooth residual plot for the time-dependent effect of the patient being a cancer patient.


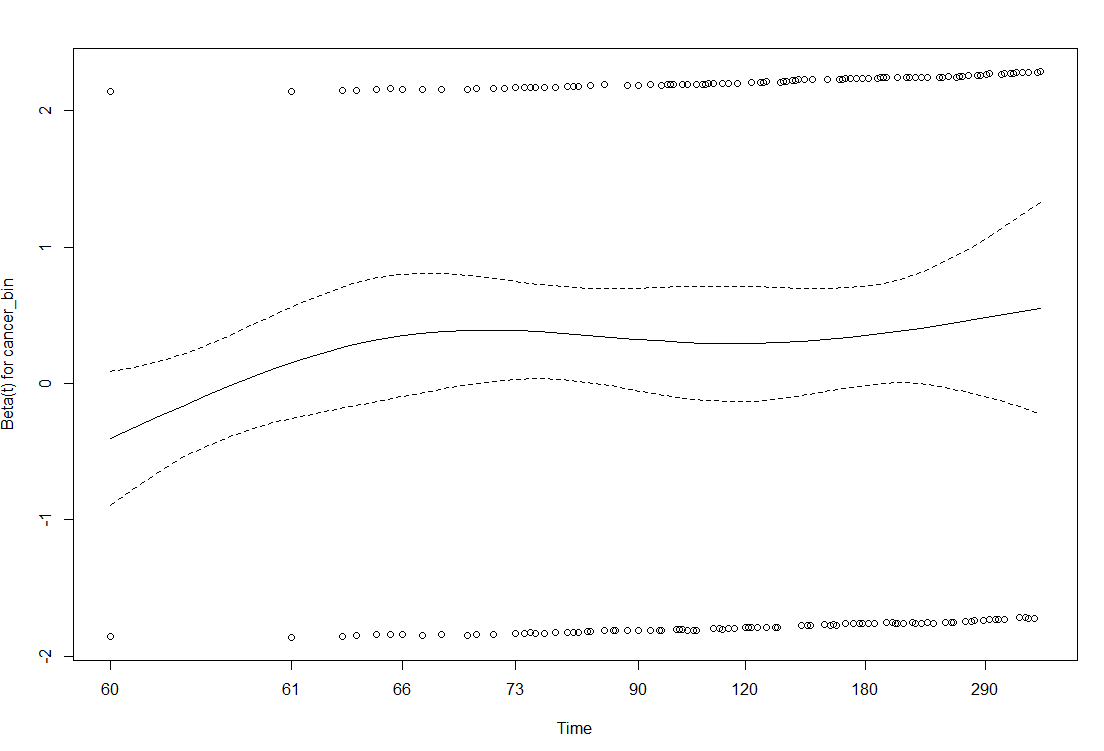


Supplemental Material E.3.

From the graph of the smooth residual plot, we see that the effect of having a cancer diagnosis is only marginally significant (p-value of 0.049). The impact of cancer on the hazard increases with time, starting with lower increases in risk two to three months following hospital discharge to slightly higher increases after three months post-discharge. The crossing hazards could explain why the effect of cancer on the risk of developing long-term use is non-significant (HR is close to 1, and the confidential intervals span the null).

Appendix E.4. Smooth residual plot for the time-dependent effect of the having a full drug co-pay status.


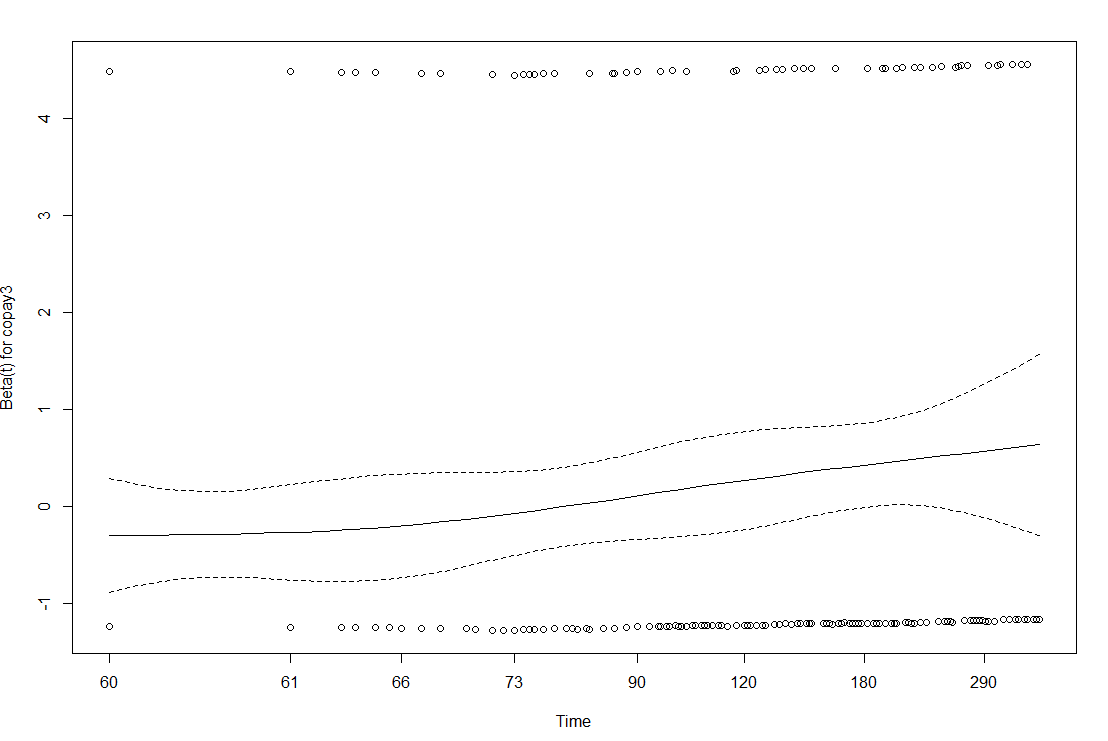


Supplemental Material E.4.

From the graph of the smooth residual plot, we see a similar delayed effect on the risk of the outcome as in having a cancer diagnosis. The graph shows that that the effect of full co-pay status is significant (p-value of 0.034). The impact of full co-pay status on the hazard increases with time, starting with lower increases in risk two to three months following hospital discharge to slightly higher increases after three months post-discharge.

Appendix F. Preliminary Power Calculations

Given that the current study relies on secondary analyses of the data from the previous trial, the sample size was determined *a priori.* Thus, we assessed the statistical power to detect clinically meaningful associations with the *a priori* fixed sample size, while accounting for the relevant empirical information. Overall, a total of 1,525 discharged patients had at least one opioid dispensation in the one year following hospital discharge. Preliminary analyses suggested that the rate of long-term opioid use in our cohort, using a cut-off of 60 days of opioid use, is around 7% and 12%. As we studied the associations of multiple potential risk factors with the development of long-term opioid use, power calculations were done by assuming a range of values for their expected prevalence, ranging from 45% for having a prescription of a strong opioid such as hydromorphone to only 4% for being admitted to the hospital due to poisoning. Under the aforementioned assumptions, we performed power calculations using the PASS program for time-to-event analyses [ref 1]. With the endpoint defined using a cutoff of 60 days to define long-term use, a Cox model-based score test at 2-tailed α=0.05, will have adequate 80% power to detect clinically important Hazard Ratio (HR) = 1.99 for rare conditions (10% prevalence) or a weaker association (HR=1.46) for a well-balanced condition with 45% prevalence. For a quantitative variable (e.g. age or number of pharmacies in the previous year), there will be 80% power to detect a moderate HR=1.23 for 1 SD increase in its value.

References:

1. Hintze J (2008). PASS 2008. NCSS, LCC. Kaysville Utah. www.ncss.com.
