# Supplementary material for: Determinants of long-term opioid use in hospitalized patients
Source: PLoS One. 2022 Dec 15;17(12):e0278992. doi: 10.1371/journal.pone.0278992 (PMC9754198; doi:10.1371/journal.pone.0278992)
Supplement: Attachment — Submitted filename: PONE-D-21-29780 Responses to Reviewers.docx [file pone.0278992.s002.docx]

| Journal Requirements: |  |
| --- | --- |
| 1. Please ensure that your manuscript meets PLOS ONE's style requirements, including those for file naming. | We have made sure the revised manuscript meets the PLOS ONE’s style requirements. |
| 2. Please ensure you have included the registration number for the clinical trial referenced in the manuscript. | We have now included the trial registration number (NCT01179867) in the manuscript. |
| 3. All PLOS journals require that the minimal data set be made fully available. If there are ethical or legal restrictions to sharing your data publicly, please explain these restrictions in detail. | We thank you for bringing this requirement to our attention. Unfortunately, due to ethical and legal restrictions, we will not be able to share the governmental provincial administrative (RAMQ) data used in these analyses publicly. Our Research Ethics Board (REB) approval for this project limits data access to Robyn Tamblyn’s team. To provide access to other researchers, they would need to obtain permission from the Institut de la Statistique du Québec for their project that would use RAMQ data. More details regarding eligibility to request data can be found here:  <https://statistique.quebec.ca/research/#/a-propos/utilisation-guichet>. To use the clinical data, they would a) have to apply for REB approval for the project, and request that a de-identified copy of the data be made available as consent was provided by patients for only Robyn Tamblyn (consent form appended). More information regarding access to data can be found here: <https://statistique.quebec.ca/research/#/demarche/etape-par-etape>. A link to submit a data request can be found here: <https://statistique.quebec.ca/zone-chercheur/#/login>. |
| 4. Please upload a copy of Figure 1, to which you refer in your text on page 7. If the figure is no longer to be included as part of the submission please remove all reference to it within the text. | We thank you for this important observation. We now have uploaded Figure 1, as a separate file. The text also refers to Figure 1 on page 7: “Figure 1 shows the breakdown of patients with respect to their previous history of opioid use, opioid administration during the hospitalization and the receipt of an opioid prescription at hospital discharge.” |
| 5. We note you have included a table to which you do not refer in the text of your manuscript. Please ensure that you refer to Table 3 in your text; if accepted, production will need this reference to link the reader to the Table. | We thank the journal for this important comment. In the newly revised version of the manuscript, we now refer to Table 3 when we present the main results of our analyses. |
| 1. An important step in fitting the multivariable Cox model is to "assess the proportional hazards assumptions". This check actually leads to handling time-varying covariates (which the authors anyway did as part of sensitivity analysis). However, I would like to see the initial checks (p-values) related to PH assessment. See the link below; testing for proportional hazards is available in various software. | We agree that this is an important step when fitting the Cox proportional hazards model. Thus, as recommended by the Reviewer, we have now tested the proportional hazards (PH) assumption and we have found that, indeed, the global p-value is significant (0.0046). In particular, we have also found that there are four individual variables (copay status, having a cancer diagnosis, having fentanyl dispensed on the first opioid prescription and having the first dose of opioid dispensed post-discharge of >90MME) for which the PH hypothesis was rejected (p<0.05 in each case). In the revised “Statistical Analyses” section, we briefly outline the methods and software used for PH testing (page 6). Given space limitations, in the “Results” section of the main manuscript we provide only the brief summary of the results of PH tests (page 8, 9). A more detailed description of the time-dependent changes in HR’s for the four variables for which the PH hypothesis was rejected is presented in section Appendix E in Supplementary Material. |
| 2. I understand this is a secondary analysis of a cluster-randomized trial. It would help the readers if a statement on sample size/power is again presented illustrating the desired effect sizes the authors wanted to achieve/start with, under the available $n$ (sample size). | We thank the Reviewer for this important comment. As now indicated at the end of the “Statistical Analyses” section (page 6 of the main manuscript), in a new Appendix F of the Supplementary Material we have added a description of power calculations, which were conducted beforehand for this study. However, please notice that, given that the current study relies on secondary analyses of the data from the previous trial, the sample size was determined *a priori*. Below, we cite the text of this section: “Given that the current study relies on secondary analyses of the data from the previous trial, the sample size was determined *a priori.* Thus, we assessed the statistical power to detect clinically meaningful associations with the *a priori* fixed sample size, while accounting for the relevant empirical information. Overall, a total of 1,525 discharged patients had at least one opioid dispensation in the one year following hospital discharge. Preliminary analyses suggested that the rate of long-term opioid use in our cohort, using a cut-off of 60 days of opioid use, is around 7% and 12%. As we studied the associations of multiple potential risk factors with the development of long-term opioid use, power calculations were done by assuming a range of values for their expected prevalence, ranging from 45% for having a prescription of a strong opioid such as hydromorphone to only 4% for being admitted to the hospital due to poisoning. Under the aforementioned assumptions, we performed power calculations using the PASS program for time-to-event analyses [ref 1]. With the endpoint defined using a cutoff of 60 days to define long-term use, a Cox model-based score test at 2-tailed α=0.05, will have adequate 80% power to detect clinically important Hazard Ratio (HR) = 1.99 for rare conditions (10% prevalence) or a weaker association (HR=1.46) for a well-balanced condition with 45% prevalence. For a quantitative variable (e.g. age or number of pharmacies in the previous year), there will be 80% power to detect a moderate HR=1.23 for 1 SD increase in its value.” |
| 3. The data is cluster-randomized; it is not clear where the "cluster" is? What are the clusters/units? | We thank the Reviewer for having pointed out the lack of clarity regarding “clusters”. The analyses of study used data on eligible patients discharged alive from two types of hospital units (medicine, surgery) and hospital location (two hospitals), resulting in a total of four possible discharge units. We now clarify in the main body of the manuscript what the main cluster is referred to. Please see also the (related) response to your point 4, below. |
| 4. It is not clear if a "frailty" proportional hazards model was fitted, which accounts for the clustering issues in cluster-randomized trials. The writeup doesn't reveal so clearly; if not considered, why? | We thank the Reviewer for this very relevant comment. In the original analyses of the manuscript, we did not account for clustering within hospital units as exploratory data had suggested no clustering within attending physician (serving as a proxy for hospital unit discharged from). However, in order to further assess the potential for possible clustering within the four discharge units, we have now fitted a frailty Cox proportional hazards model, that accounted for clustering within the four clusters mentioned above and assessed the importance of potential clustering. The methods of frailty analyses are now briefly outlined near the end of the “Sensitivity Analyses” subsection of Statistical Analyses (page 7) of the main manuscript). Results (briefly mentioned in the “Results” section of the revised manuscript (page 9), showed a completely non-significant p-value for the clustering effect: p=0.94, confirming the lack of evidence of clustering in our study cohort. This may be partly explained by the fact that we adjust for (important and statistically significant) the fixed effect of medical vs surgical units. |

References:

1. Hintze J (2008). PASS 2008. NCSS, LCC. Kaysville Utah. www.ncss.com.
